# Aortic Root Geometry and Valve Competence After Aortic Valve Neocuspidization: Insights From the Sinotubular Junction-to-Annulus Ratio

**DOI:** 10.1093/icvts/ivag182

**Published:** 2026-07-07

**Authors:** Hirofumi Kasahara, Yoshito Inoue, Koji Funaishi, Sohsyu Kotani

**Affiliations:** Department of Cardiovascular Surgery, Hiratsuka City Hospital, Kanagawa, 254-0065, Japan; Tokushukai Hanyu General Hospital, Saitama, 348-8505, Japan; Department of Cardiovascular Surgery, Hiratsuka City Hospital, Kanagawa, 254-0065, Japan; Department of Cardiovascular Surgery, Tokai University School of Medicine, Kanagawa, 259-1193, Japan

**Keywords:** aortic regurgitation, aortic root geometry, aortic valve reconstruction, Ozaki procedure, sinotubular junction

## Abstract

**Objectives:**

Aortic valve neocuspidization, or the Ozaki procedure, is an alternative to prosthetic valve replacement. However, the association between native aortic root geometry, particularly the sinotubular junction-to-annulus ratio, and postoperative valve competence remains unclear. This study was designed to investigate the association between the sinotubular junction-to-annulus ratio and postoperative aortic regurgitation severity.

**Methods:**

We retrospectively analysed 139 patients who underwent aortic valve neocuspidization between 2012 and 2023. At 1 year, the patients were stratified into either moderate or less-than-moderate aortic regurgitation groups. Intraoperative sinotubular junction and annulus diameters were measured using transoesophageal echocardiography. Logistic regression and receiver operating characteristic analyses were performed.

**Results:**

At 1 year, 11 (7.9%) patients developed moderate aortic regurgitation, while 128 (92.1%) had less-than-moderate aortic regurgitation. Patients with moderate aortic regurgitation had higher sinotubular junction-to-annulus ratios (moderate group: median, 1.40; less-than-moderate group: median, 1.19). Receiver operating characteristic analysis showed excellent discrimination, with an optimal cut-off of 1.30. Each 0.1-unit increase in the ratio was associated with a 3.65-fold increase in the risk of developing moderate aortic regurgitation. Preoperative aortic regurgitation severity was associated with univariable analysis outcomes but lost significance after adjustment for the sinotubular junction ratio.

**Conclusions:**

An increased sinotubular junction-to-annulus ratio may be associated with the development of postoperative aortic regurgitation after aortic valve neocuspidization. These findings suggest that the geometric association between the sinotubular junction and the annulus may play a role in valve competence after aortic valve neocuspidization.

## Graphical abstract

**Figure:**
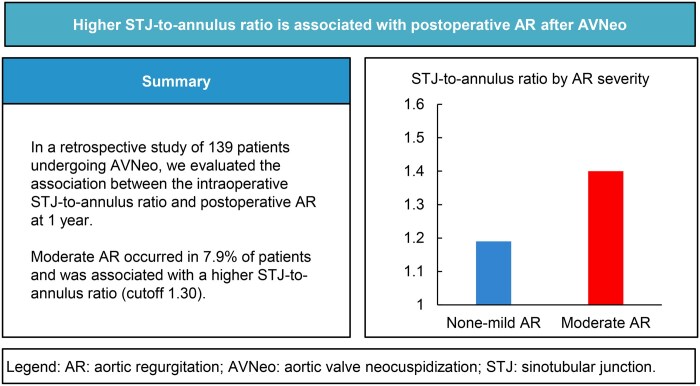

## INTRODUCTION

Aortic valve neocuspidization (AVNeo), also referred to as the Ozaki procedure, has emerged as an alternative to conventional prosthetic valve replacement for the treatment of aortic valve disease.^1–3^ This procedure involves reconstruction of the aortic valve using glutaraldehyde-treated autologous pericardium to create anatomically tailored neocusps that closely replicate native valve morphology and function.^4^^,^^5^ Through replicating native valve dynamics and avoiding prosthetic materials, AVNeo could mitigate the inherent limitations of conventional valve replacements. Although AVNeo generally provides excellent haemodynamic outcomes,^3^^,^^6^ a subset of patients has been reported to develop postoperative aortic regurgitation (AR), raising concerns regarding valve competence.^6^^,^^7^ Contemporary concepts of aortic valve repair emphasize that valve competence depends not only on cusp morphology but also on the geometric configuration of the functional aortic annulus, which includes the ventriculo-aortic junction, sinuses of Valsalva, and sinotubular junction (STJ).^8–10^ Unlike stented bioprosthetic replacements, the haemodynamic performance of AVNeo may be influenced by the native geometry of the aortic root, particularly the proportional associations among the annulus, the sinus of Valsalva, and the STJ.^8^^,^^10^ Among these geometric parameters, the STJ-to-annulus ratio may be specifically relevant for cusp coaptation in AVNeo; however, its prognostic value remains uncertain.

This study was designed to evaluate the association between the intraoperative STJ-to-annulus ratio and the development of moderate-or-greater AR 1 year after AVNeo. Clarifying the relative value of these factors may help optimize patient selection, refine surgical strategies, and support the long-term durability of AVNeo.

## METHODS

### Study population and design

Between 2012 and December 2023, a total of 167 patients underwent AVNeo at Hiratsuka City Hospital. The study population comprised patients with aortic valve disease who required an operation, including aortic stenosis and AR. The indications for AVNeo evolved over the study period. Initially, the procedure was performed primarily for patients with a small aortic annulus (≤21.5 mm). Following favourable clinical outcomes, the indication was gradually expanded to include a broader spectrum of aortic valve disease.

Concomitant procedures were performed as indicated. Patients who underwent reconstruction with xenopericardium or an operation for active infective endocarditis were excluded, resulting in 158 eligible study patients. Of these, 139 had complete intraoperative transoesophageal echocardiography (TEE) measurements and 1-year transthoracic echocardiography follow-up data, forming the study cohort.

This study was conducted in accordance with the principles of the Declaration of Helsinki and approved by the institutional review board of Hiratsuka City Hospital (approval number: 25–002; date: 21 June 2023). The requirement for written informed consent was waived owing to the study’s retrospective design. All data were anonymized prior to analysis. No data or biological material was collected or stored for multiple or indefinite future use; therefore, the requirements of the World Medical Association Declaration of Taipei were not applicable to this study. Preoperative and postoperative clinical data, including baseline demographic variables, valve pathology, left ventricular (LV) dimensions, and concomitant procedures, were obtained from the institutional databases and medical records.

### Surgical procedure

The surgical procedure was performed according to that originally described by Ozaki et al.^2^^,^^3^ Following a median sternotomy, autologous pericardium was harvested and treated with 0.6% glutaraldehyde for 10 min. After weaning the patient from cardiopulmonary bypass, intraoperative TEE was used to measure annular (hinge-to-hinge) and STJ (inner edge-to-inner edge) diameters at mid-systole. Aortic valve morphology was assessed by intraoperative inspection and classified as tricuspid or bicuspid. The diameter of the ascending aorta was measured on preoperative computed tomography. During the early study period, concomitant ascending aortic replacement was performed when the diameter was ≥50 mm. From 2019 onward, the surgical indication was expanded to include patients with a diameter ≥40 mm. In these cases, the ascending aorta was transected approximately 15 mm above the STJ, where the proximal anastomosis was constructed. In principle, a 24-mm prosthetic graft was used to achieve STJ stabilization; however, 26- and 28-mm grafts were used in 2 early cases.

### Echocardiographic assessment and follow-up

Preoperative AR was graded as none, mild, moderate, or severe, in accordance with American Society of Echocardiography recommendations^11^ and subsequently consolidated into 3 categories, namely, none (including trace), mild, and moderate-or-greater (including severe), for statistical analysis. Postoperative AR was dichotomized as moderate or greater versus less than moderate. The primary outcome was the presence of moderate-or-greater AR at 1 year.

### Statistical analysis

Continuous variables are presented as medians (IQR) and categorical variables, as numbers (percentage). Given the limited sample size of the moderate AR group, comparisons were performed using the Mann–Whitney *U* or the Fisher exact test. Multivariable logistic regression included the preoperative AR grade (3 categories) and the intraoperative STJ-to-annulus ratio (continuous). Odds ratios for the STJ ratio were recalculated per 0.1-unit increment to facilitate clinical interpretability. The receiver operating characteristic (ROC) analysis was used to assess discriminatory performance; the area under the curve (AUC) was calculated, cut-off values were determined using the Youden index, and predictive values were calculated. Associations between the preoperative AR grade and the STJ ratio were evaluated using the Spearman correlation and the Kruskal–Wallis test. All statistical analyses were performed using SPSS version 26.0 (IBM Corp.). A 2-sided *P-*value <.05 was considered statistically significant. Given the limited number of outcome events, the present analysis should be considered exploratory in nature.

## RESULTS

### Patient characteristics

Baseline clinical and echocardiographic characteristics of the overall cohort, along with comparisons according to postoperative AR severity at 1 year, are summarized in **Table 1**. Among the 139 patients with complete 1-year follow-up data, 11 (7.9%) developed moderate AR, whereas 128 (92.1%) had less-than-moderate AR. One patient required an early reoperation for infective endocarditis and was excluded from the outcome analysis.

**Table 1.** Baseline Characteristics of the Study Cohort and Stratification According to Postoperative Aortic Regurgitation Severity at 1 Year

Postoperative AR	None-to-mild AR	Moderate AR	*P*-value
	*n* = 128	*n* = 11	
Age, years	75 [70-79]	75 [68-77]	.570
Male sex, *n* (%)	60 (46.9)	7 (63.6)	.537
BSA, m^2^	1.54 [1.4-1.67]	1.55 [1.42-1.65]	.993
**Cardiovascular risk factors**			
Hypertension, *n* (%)	94 (73.4)	10 (90.9)	.290
Hyperlipidaemia, *n* (%)	58 (45.3)	6 (54.5)	.750
Diabetes mellitus, *n* (%)	39 (30.5)	2 (18.2)	.507
Haemodialysis, *n* (%)	8 (6.3)	0	1.000
Post PCI, *n* (%)	9 (7.0)	1 (9.1)	.574
EuroSCORE (logistic)	1.82 [1.38-2.42]	1.48 [1.33-2.25]	.419
**Preoperative echocardiogram (TTE)**			
EF, %	66.6 [56.6-74]	63.9 [46.1-71.6]	.141
LVDd, mm	49.9 [40.9-51.1]	53.0 [50.1-62.5]	**.009**
LVDs, mm	28.9 [24.9-36.6]	40.0 [30-43.4]	**.034**
LAD, mm	40.7 [37-45]	39.6 [32.5-42.7]	.564
Max PG, mmHg	68.5 [49-96]	44.0 [18.3-84.5]	.103
Mean PG, mmHg	40 [27.5-56]	21.9 [9.5-50.5]	.165
AVA, cm^2^	0.7 [0.5-0.8]	0.7 [0.5-1.9]	.845
**AR grade, *n* (%)**			**.040**
None	21 (16.4)	2 (18.2)	
Mild	72 (56.3)	1 (9.1)	
Moderate or greater	35 (27.3)	8 (72.7)	
**Preoperative valve morphology**			.059
Tricuspid valve, %	103 (80.4)	6 (54.5)	
Bicuspid valve, %	25 (19.5)	5 (45.5)	
**Ascending aortic diameter on CT**			
Diameter, mm	38.2 [35.4-40.9]	42.2 [40.2-45.2]	**.002**
Diameter >40 mm, *n* (%)	42 (32.8)	8 (72.7)	**.017**

Baseline characteristics of the overall cohort are presented, with additional stratification according to postoperative aortic regurgitation severity at 1 year. Preoperative aortic regurgitation was categorized as none, mild, and moderate or greater (including severe) according to guideline-based grading. Percentages are presented within each postoperative aortic regurgitation group. Values are expressed as median (interquartile range [IQR]) for continuous variables and number (percentage) for categorical variables. Bold values indicate statistically significant differences (P < 0.05).

Abbreviations: AR, aortic regurgitation; AVA, aortic valve area; BSA, body surface area; CT, computed tomography; EF, ejection fraction; LAD, left atrial diameter; LVDd, left ventricular end-diastolic diameter; LVDs, left ventricular end-systolic diameter; PCI, percutaneous coronary intervention; PG, pressure gradient; TTE, transthoracic echocardiogram.

Patients were stratified according to AR severity at 1 year postoperatively (**Table 1**). Patients with postoperative moderate AR had significantly larger LV dimensions than those with less-than-moderate AR. Postoperative moderate AR also occurred more frequently in patients with preoperative moderate-or-greater AR (*P *= .040). In contrast, patients in the none-to-mild postoperative AR group were predominantly those with aortic stenosis, whose preoperative AR was limited to a severity level of trivial/mild. These patients tended to have smaller LV dimensions and higher pressure gradients; however, the differences did not reach statistical significance. Operative variables were comparable between the groups (**Table 2**).

**Table 2.** Operative Variables According to the Severity of Postoperative Aortic Regurgitation Severity at 1 Year

Postoperative AR	None-to-mild AR	Moderate AR	*P*-value
	*n* = 128	*n* = 11	
Clamp time, min	134 [103-165]	124 [105-143]	.242
CPB time, min	174 [137-211]	161 [112-210]	.248
Ascending aortic replacement, *n* (%)	22 (17.2)	2 (18.1)	1.000
CABG, *n* (%)	21 (16.4)	1 (9.1)	1.000
Valve operation^a^, *n* (%)	10 (7.8)	0	.590
Operation for AF	10 (7.8)	0	.590

Values are expressed as median [IQR] for continuous variables and number (percentage) for categorical variables.

a Valve surgery includes mitral or tricuspid valve procedures. Some patients underwent more than 1 concomitant procedure.

Abbreviations: AF, atrial fibrillation; AR, aortic regurgitation; CABG, coronary artery bypass grafting; CPB, cardiopulmonary bypass; IQR, interquartile range.

### Preoperative anatomical factors and postoperative AR

Preoperative bicuspid aortic valve morphology was associated with a higher incidence of postoperative moderate AR than was tricuspid valve morphology (16.7% vs 5.5%, respectively). Statistical significance was not reached (*P *= .059), but these findings suggest a possible association between bicuspid valve morphology and postoperative moderate AR (**Table 1**).

Patients with a preoperative ascending aortic diameter ≥40 mm had a significantly higher incidence of postoperative moderate AR than those with a diameter <40 mm (16.0% vs 3.4%, *P *= .017, **Table 1**).

Among the 50 patients with a preoperative ascending aortic diameter ≥40 mm, postoperative moderate AR occurred in 6 of 26 patients (23.1%) who did not undergo ascending aortic replacement and in 2 of 24 patients (8.3%) who underwent concomitant replacement. This difference did not reach statistical significance (*P *= .25), but postoperative AR tended to occur less frequently in patients undergoing ascending aortic replacement.

### STJ-to-annulus ratio and postoperative AR

**Figure 1.**
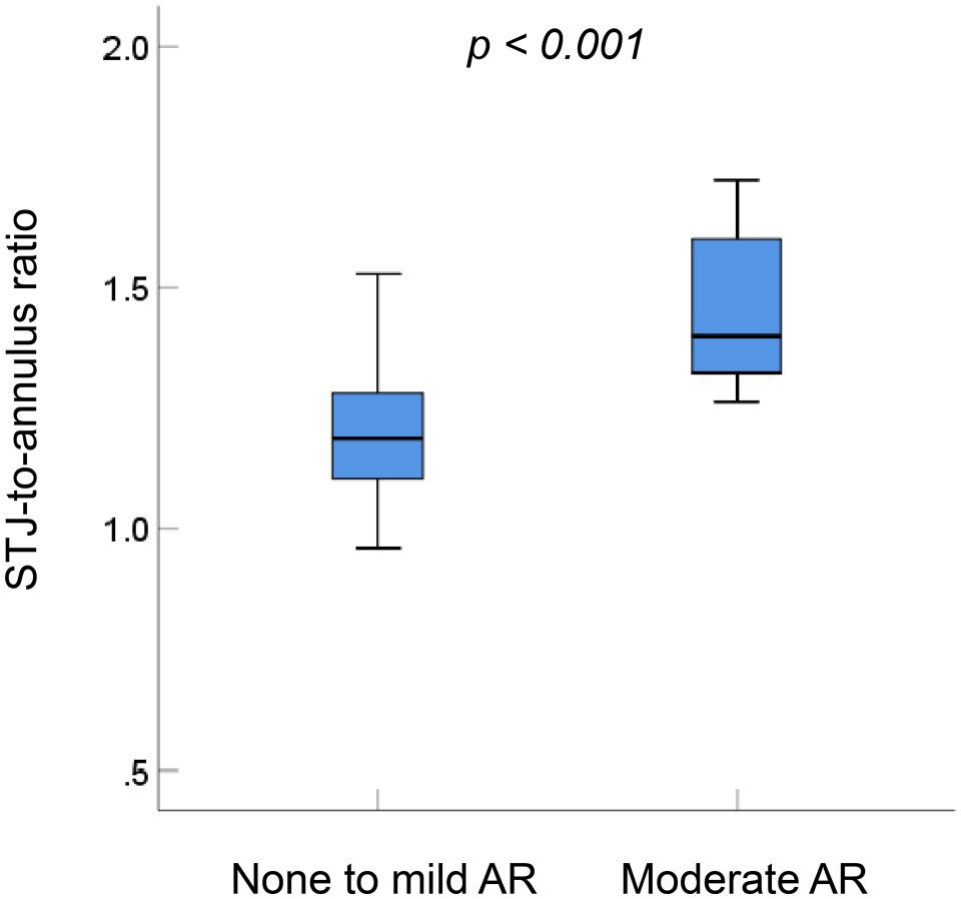
Distribution of the Intraoperative Sinotubular Junction-to-Annulus Ratio According to Postoperative Aortic Severity at 1 Year. Patients with postoperative moderate aortic regurgitation had significantly higher ratios than those with less-than-moderate aortic regurgitation (median 1.40 vs 1.19; *P* < .001). Boxes represent interquartile ranges; horizontal lines indicate medians; and whiskers denote the minimum and maximum values excluding outliers.

The intraoperative STJ-to-annulus ratio, measured using TEE after weaning the patient from cardiopulmonary bypass, was significantly higher in patients who developed moderate AR (median, 1.40 [1.32-1.60]) than in those with less-than-moderate AR (1.19 [1.10-1.28], *P *< .001) (**Table 3**, **Figure 1**). Results of the univariate logistic regression analysis showed that the STJ-to-annulus ratio was significantly associated with postoperative moderate AR (*P *< .001) (**Table 4**).

**Table 3.** Intraoperative Echocardiographic Measurements and Postoperative Aortic Regurgitation Severity

Postoperative AR	None-to-mild AR	Moderate AR	*P*-value
	*n* = 128	*n* = 11	
Annulus, mm	20 [18-22]	19 [18-20]	.237
STJ, mm	23 [22-26]	28 [24-32]	**.030**
STJ/annulus ratio	1.19 [1.10-1.28]	1.40 [1.32-1.60]	**<.001**

Values are presented as median [IQR].

Comparisons between groups were performed using a Mann–Whitney *U* test. Bold values indicate statistically significant differences (*P* < 0.05).

Abbreviations: AR, aortic regurgitation; IQR, interquartile range; STJ, sinotubular junction.

**Table 4.** Multivariable Logistic Regression Analysis of Factors Associated with Postoperative Moderate Aortic Regurgitation at 1 Year

Variable	OR (95% CI)	*P-*value
STJ-to-annulus ratio (per 0.1 increase)	3.65 (1.83-7.27)	**<.001**
Preoperative AR severity (ref. = none)		
Mild vs none	0.14 (0.01-2.17)	.157
Moderate-or-greater vs none	1.72 (0.20-14.78)	.620

Values are expressed as ORs with 95% CIs. The STJ-to-annulus ratio is expressed per 0.1-unit increments. Preoperative AR severity was modelled using a 3-grade classification system (ref. = none). Bold values indicate statistically significant differences (*P* < 0.05).

Abbreviations: AR, aortic regurgitation; OR, odds ratio; ref., reference; STJ, sinotubular junction.

### Discriminatory performance of the STJ-to-annulus ratio

**Figure 2.**
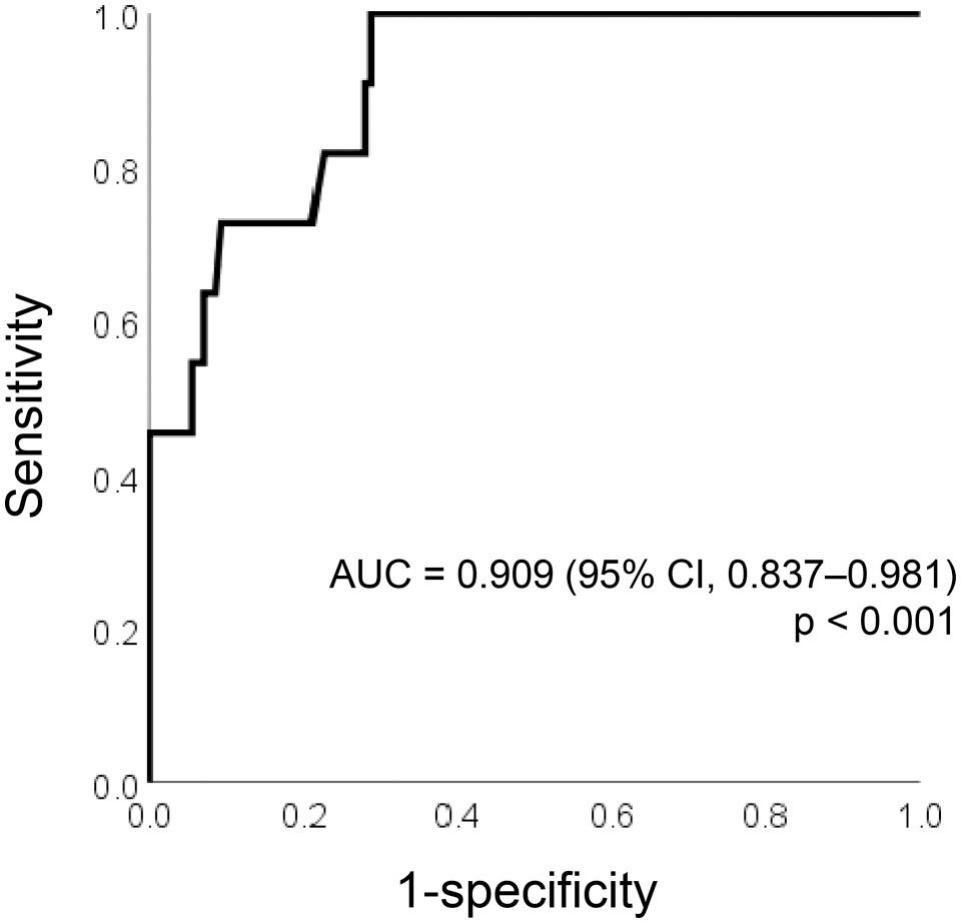
Receiver Operating Characteristic Curve of the Intraoperative Sinotubular Junction-to-Annulus Ratio for Postoperative Moderate-or-Greater Aortic Regurgitation at 1 Year. The area under the curve was 0.909 (95% CI, 0.837-0.981; *P* < .001), indicating good discriminatory performance. The optimal cut-off value of 1.30 yielded a sensitivity of 72.7% and a specificity of 79.7%.

The ROC analysis demonstrated good discriminatory performance of the STJ-to-annulus ratio for moderate AR at 1 year (AUC, 0.909; 95% CI, 0.837-0.981; *P *< .001) (**Figure 2**). The optimal cut-off for the STJ-to-annulus ratio was 1.30, yielding a sensitivity of 72.7% and a specificity of 79.7%. At this threshold, the positive predictive value was modest (22.9%), but the negative predictive value was high (97.0%). Accordingly, a ratio below 1.30 was associated with a low likelihood of moderate-or-greater AR at 1 year, whereas a ratio ≥1.30 identified patients at increased risk, though with limited positive predictive accuracy.

### Association between preoperative and postoperative AR

**Figure 3.**
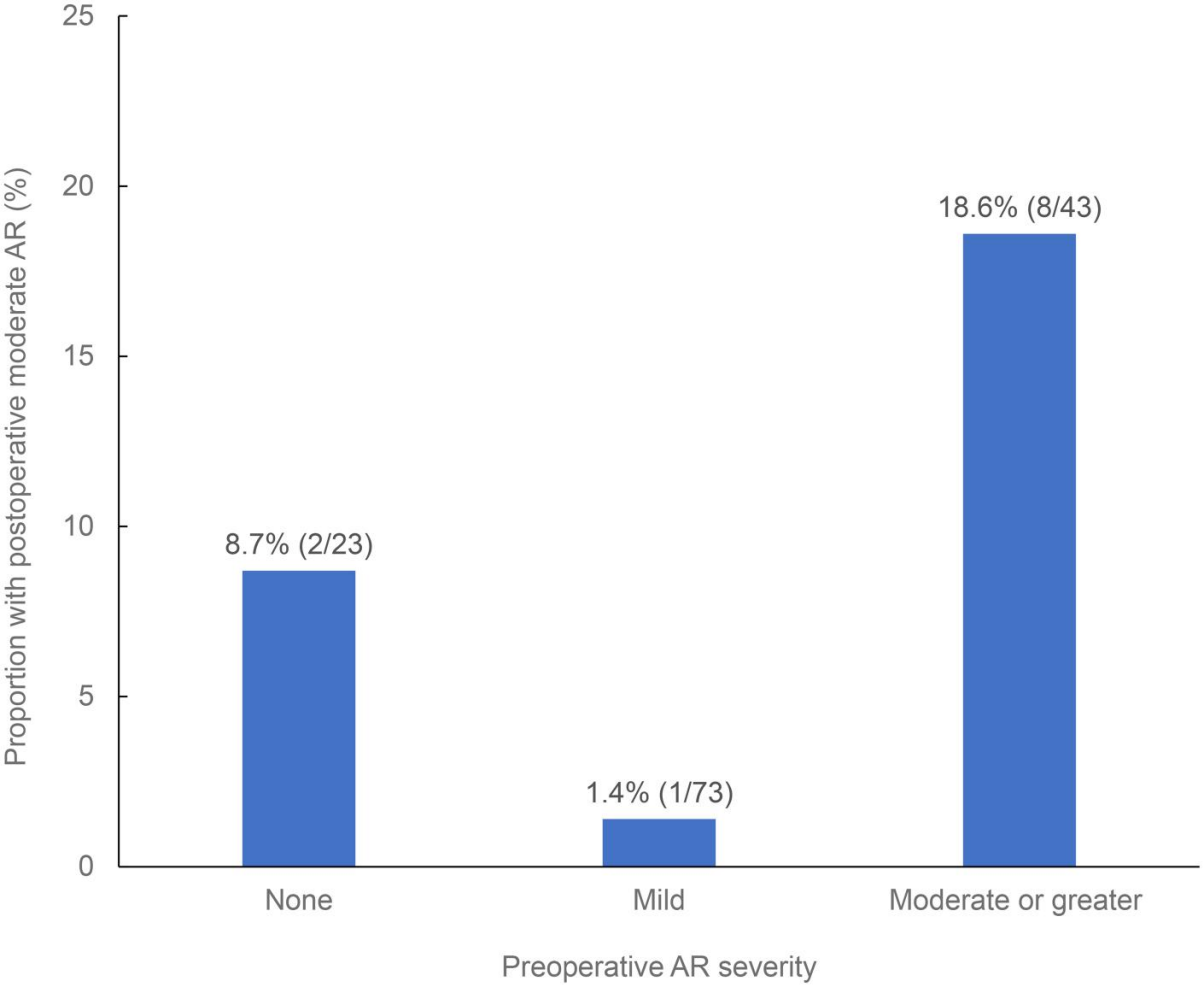
Incidence of Postoperative Moderate Aortic Regurgitation at 1 Year According to Preoperative Aortic Regurgitation Severity. Rates of postoperative moderate aortic regurgitation increased stepwise across categories: 8.7% (2/23) in patients with none, 1.4% (1/73) in those with mild aortic regurgitation, and 18.6% (8/43) in those with moderate or greater preoperative aortic regurgitation (*P* = .004, *χ*^2^ test).

Preoperative AR severity was associated with postoperative moderate AR (*P *= .004, *χ*^2^ test; *P* for trend = .040) (**Figure 3**, **Table S1**). However, no significant association was observed between preoperative AR grade and the intraoperative STJ-to-annulus ratio (Kruskal–Wallis test, *P *= .36). Spearman’s rank correlation analysis likewise showed no significant association (*ρ*  =  0.09, *P *= .29), confirming the absence of correlation between the 2 variables.

### Multivariable model

Univariable logistic regression analysis showed that preoperative AR severity was associated with postoperative outcomes (*P *= .04). However, this effect lost significance after adjusting for the STJ-to-annulus ratio. After adjustment, the STJ-to-annulus ratio remained significantly associated with postoperative moderate AR (odds ratio per 0.1-unit increment, 3.65; 95% CI, 1.83-7.27; *P *< .001) (**Table 4**). The model calibration and explanatory power were acceptable (Hosmer–Lemeshow *P *= .957; Nagelkerke *R*^2^ = 0.556).

Additional clinical follow-up data beyond 1 year was available only for a subset of patients, and these data are presented in **Material S2**. These data were not included in the prespecified primary analysis.

## DISCUSSION

In this study, the intraoperative STJ-to-annulus ratio was found to be associated with the occurrence of moderate AR 1 year after AVNeo. Each 0.1-unit increment in the ratio was associated with an odds ratio of 3.65 for postoperative AR. The ROC analysis identified a cut-off value of approximately 1.30, above which the incidence of postoperative moderate AR increased. Although the number of events in the present cohort was limited, these findings suggest that the geometric association between the STJ and annulus may influence valve competence after cusp reconstruction.

From a biomechanical standpoint, enlargement of the STJ alters commissural geometry, reduces central leaflet coaptation, and concentrates stress at the commissures, thereby predisposing the patient to central regurgitation.^8^^,^^9^ This mechanism corresponds to a type I lesion in the functional classification of AR proposed by El Khoury et al., in which dilation of the functional aortic annulus—particularly at the STJ—impairs cusp coaptation.^8^^,^^10^^,^^12^ Previous echocardiographic studies based on transthoracic measurements have shown that the STJ is normally slightly larger than the aortic annulus, with reported STJ-to-annulus ratios generally ranging from approximately 1.15 to 1.32.^13^^,^^14^ Enlargement beyond this physiological association may attenuate inward-directed closing forces and predispose the patient to leaflet malcoaptation and regurgitation.^8^^,^^9^ The cut-off point identified here is broadly consistent with this reported geometric association.

Unlike rigid stented prostheses, AVNeo involves the use of autologous pericardial leaflets directly sutured to the aortic annulus, thereby preserving annular compliance.^4^^,^^5^ This anatomical reconstruction allows physiologic expansion during systole and attenuates closing shock in diastole, thereby promoting favourable haemodynamics and supporting long-term durability through reduced cusp stress.^3^^,^^4^^,^^6^

However, this flexibility may render the construct vulnerable to geometric perturbations when the STJ is dilated. Modern concepts of aortic valve repair emphasize that valve competence depends on the geometric balance of the functional aortic annulus.^8–10^^,^^15^ Our findings extend this concept by showing that STJ dilatation predisposes patients to moderate AR after AVNeo, which is an early marker of geometric vulnerability rather than of symptomatic or severe disease.

In the present cohort, patients with a preoperative ascending aortic diameter ≥40 mm showed a significantly higher incidence of postoperative moderate AR than those with smaller diameters. This finding suggests that dilation of the ascending aorta may reflect geometric alterations extending to the STJ. However, the STJ-to-annulus ratio, rather than the absolute aortic diameter, is reported to be independently associated with postoperative AR.^13^ These findings indicate that ascending aortic dilation may act as a surrogate marker of geometric distortion at the level of the STJ rather than as a direct contributor of valve competence.

### Preoperative AR and aortic root geometry

Preoperative AR is typically associated with root dilatation; however, this correlation was not observed in our study.^16^ A likely explanation is that surgical manipulations near the STJ, such as an aortotomy performed 15 mm above the right coronary ostium or concomitant ascending aortic graft replacement, reduced the apparent STJ diameter, resembling the intentional downsizing reported by Al-Atassi et al.^17^ in bicuspid AR, in which graft replacement decreased the STJ by approximately 9 mm on average. These findings support the understanding that intraoperative measurements obtained after aortic root reconstruction may better reflect the functional geometry of the aortic root.

Additional exploratory analyses suggested a potential role of aortic root configuration. Although the difference did not reach statistical significance (*P* = .25), the lower incidence of postoperative AR observed in patients undergoing ascending aortic replacement may be consistent with the concept that stabilization of the STJ improves the STJ-to-annulus association.^15^ This interpretation aligns with findings of surgical studies demonstrating that restoration of STJ geometry enhances leaflet coaptation and valve competence after aortic valve repair.^10^^,^^18^^,^^19^

We also observed a higher proportion of postoperative moderate AR among patients with bicuspid aortic valves compared with those with tricuspid valves. Bicuspid valve disease is frequently associated with abnormalities of aortic root geometry.^20^ Previous studies have demonstrated that such geometric asymmetry is associated with increased valve dysfunction and less favourable repair durability.^20^ These structural characteristics may predispose patients to geometric imbalance after AVNeo.

Ozaki et al.^3^ reported favourable midterm outcomes in a series of 850 patients, including 254 with AR. Their large clinical series demonstrated the broad applicability of AVNeo across diverse pathologies, supported by technical refinements. In bicuspid valves, they also performed external reinforcement at the commissural level with a felt strip to reduce the risk of future dilatation.^2^^,^^3^^,^^21^

The clinical implications of our findings remain exploratory; however, careful assessment of aortic root geometry may be warranted in patients with an enlarged STJ compared with the annulus, particularly in those with preoperative AR, ascending aortic dilatation, or bicuspid valve morphology.^4^^,^^13^^,^^15^^,^^19^ In such situations, strategies aimed at restoring a balanced STJ-to-annulus association may theoretically improve postoperative valve competence after AVNeo. However, the present study was not designed to evaluate specific operative strategies, and further studies are required.

### Limitations

This study has some limitations that should be acknowledged. It was a retrospective, single-centre analysis, and the number of outcome events was small. Consequently, the multivariable model may be susceptible to overfitting, and the estimated effect size of the STJ-to-annulus ratio as an independent predictor should be interpreted with caution. Further, the follow-up duration was limited to 1 year for the primary analysis. Preoperative STJ-to-annulus ratios were not systematically available, which limits the ability to assess their predictive value. The potential influence of surgical experience and technical variability was not examined.^3^^,^^7^ Lastly, other anatomical determinants were not included in our analysis. Larger multicentre studies with longer follow-up periods are required to validate and expand these findings.

## CONCLUSION

The present findings suggest that the geometric association between the STJ and the annulus may be associated with postoperative valve competence after AVNeo. Although confirmation in larger cohorts with longer follow-up is required, our findings support the understanding that aortic root geometry may play a role in the durability of reconstructed aortic valves using autologous pericardium.

## Supplementary Material

ivag182_Supplementary_Data

## Data Availability

The data underlying this article cannot be shared publicly due to patient privacy and institutional confidentiality policies. The data may be shared on reasonable request to the corresponding author, subject to institutional approval. The data underlying this study are available in this article and in its online supplementary material.

## GENERATIVE AI AND AI-ASSISTED TECHNOLOGIES

During the preparation of this work, the authors used ChatGPT for assistance with English translation. After using this tool/service, the authors reviewed and edited the content as needed and take full responsibility for the content of the published article, including the analysis of statistical data using AI tools.

## ABBREVIATIONS

AR: aortic regurgitation
AUC: area under the curve
AVNeo: aortic valve neocuspidization
LV: left ventricular
ROC: receiver operating characteristic
STJ: sinotubular junction
TEE: transoesophageal echocardiography
