# Supplementary material for: Aortic Root Geometry and Valve Competence After Aortic Valve Neocuspidization: Insights From the Sinotubular Junction-to-Annulus Ratio
Source: Interdiscip Cardiovasc Thorac Surg. 2026 Jul 7;41(7):ivag182. doi: 10.1093/icvts/ivag182 (PMC13385336; doi:10.1093/icvts/ivag182)
Supplement: ivag182_Supplementary_Data [file ivag182_supplementary_data.zip › Supplementary Table S1.docx]

**Supplementary Table S1.** Relationship between preoperative AR severity and moderate AR at one year postoperatively.

| **Preoperative AR severity** | **None-to-mild**  **postoperative AR, n (%)** | **Moderate**  **postoperative AR, n (%)** |
| --- | --- | --- |
| None (n=23) | 21 (91.3) | 2 (8.7) |
| Mild (n=73) | 72 (98.6) | 1 (1.4) |
| Moderate or greater (n=43) | 35 (81.4) | 8 (18.6) |
| **Total (n=139)** | 128 (92.1) | 11 (7.9) |

Values are presented as numbers (percentages). Pearson’s χ² test demonstrated a significant association (χ² = 11.05, df = 2, p = 0.004; Cramér’s V = 0.282). A stepwise increase in postoperative moderate AR was observed across preoperative AR grades (p for trend = 0.040). AR, aortic regurgitation.
