# Supplementary material for: Aortic Root Geometry and Valve Competence After Aortic Valve Neocuspidization: Insights From the Sinotubular Junction-to-Annulus Ratio
Source: Interdiscip Cardiovasc Thorac Surg. 2026 Jul 7;41(7):ivag182. doi: 10.1093/icvts/ivag182 (PMC13385336; doi:10.1093/icvts/ivag182)
Supplement: ivag182_Supplementary_Data [file ivag182_supplementary_data.zip › Supplementary_Table_S2.docx]

**Supplementary Table S2.** Additional follow-up of patients with moderate AR at 1 year

Of 11 patients who had developed moderate AR at the 1-year evaluation, additional follow-up data were obtained (median follow-up: 83 months, interquartile range, 61–105). One patient progressed to severe AR and underwent surgical aortic valve replacement at another institution 68 months after the initial operation. No marked cusp degeneration was observed, and commissural structural failure was considered the primary mechanism. The remaining 10 patients are currently under follow-up. Of these, three patients had shown progression in AR severity (moderate-to-severe), whereas the other seven patients had remained clinically stable without requiring reintervention.

| Patient No. | Sex | Age, years | AR at 1 year | Follow-up | Final AR grade | Mean PG | LVEF | LV dimensions | Reintervention |
| --- | --- | --- | --- | --- | --- | --- | --- | --- | --- |
|  |  |  |  | (months) |  | (mmHg) | (%) | LVDd/LVDs (mm) |  |
| 1 | M | 79 | moderate | 147 | moderate-to-severe | 12.4 | 65 | 53/34 | - |
| 2 | M | 50 | moderate | 68 | severe | 5.1 | 54 | 57/41 | AVR |
| 3 | F | 75 | moderate | 70 | moderate | 7.4 | 67 | 51/32 | - |
| 4 | M | 80 | moderate | 83 | moderate | 11.6 | 72 | 49/28 | - |
| 5 | F | 70 | moderate | 91 | mild | 15.6 | 71 | 48/29 | - |
| 6 | M | 76 | moderate | 90 | mild-to-moderate | 6.3 | 56 | 47/34 | - |
| 7 | F | 61 | moderate | 62 | moderate-to-severe | 7.9 | 46 | 51/39 | - |
| 8 | M | 75 | moderate | 83 | mild-to-moderate | 4.8 | 55 | 52/37 | - |
| 9 | M | 68 | moderate | 84 | moderate-to-severe | 5.7 | 67 | 49/31 | - |
| 10 | M | 69 | moderate | 66 | mild | 4.9 | 69 | 48/29 | - |
| 11 | F | 78 | moderate | 71 | moderate | 10.7 | 74 | 42/24 | - |

Abbreviations: AR, aortic regurgitation; AVR, aortic valve replacement; F, female; LVEDD/LVESD, left ventricular diastolic and systolic diameters; LVEF, left ventricular ejection function; M, male; No., number; PG, pressure gradient
